# Reprogramming of glutamine metabolism via glutamine synthetase silencing induces cisplatin resistance in A2780 ovarian cancer cells

**DOI:** 10.1186/s12885-021-07879-5

**Published:** 2021-02-17

**Authors:** Jing Guo, Kiyotoshi Satoh, Sho Tabata, Masaru Mori, Masaru Tomita, Tomoyoshi Soga

**Affiliations:** 1 Institute for Advanced Biosciences, Keio University, 246-2 Mizukami, Kakuganji, Tsuruoka, 997-0052 Japan; 2 Graduate School of Media and Governance, Keio University, 5322 Endo, Fujisawa, 252-0882 Japan; 3 Faculty of Environment and Information Studies, Keio University, 5322 Endo, Fujisawa, 252-0882 Japan

**Keywords:** Ovarian cancer, Cisplatin resistance, Glutamine synthetase, Metabolome, CE-TOFMS

## Abstract

**Background:**

Cisplatin (CDDP) significantly prolongs survival in various cancers, but many patients also develop resistance that results in treatment failure. Thus, this study aimed to elucidate the underlying mechanisms by which ovarian cancer cells acquire CDDP resistance.

**Methods:**

We evaluated the metabolic profiles in CDDP-sensitive ovarian cancer A2780 cells and CDDP-resistant A2780cis cells using capillary electrophoresis-time-of-flight mass spectrometry (CE-TOFMS). We further examined the expression of glutamine metabolism enzymes using real-time PCR and Western blot analyses. Cell viability was accessed using 3-(4,5-dimethylthiazol-2-yl)-2,5-diphenyltetrazolium bromide (MTT) assay.

**Results:**

The results showed that levels of glutamine, glutamate, and glutathione (GSH), a key drug resistance mediator synthesized from glutamate, were significantly elevated in A2780cis cells than those in A2780 cells. Furthermore, glutamine starvation decreased the GSH levels and CDDP resistance in A2780cis cells. Interestingly, the expression of glutamine synthetase (GS/GLUL), which synthesizes glutamine from glutamate and thereby negatively regulates GSH production, was almost completely suppressed in resistant A2780cis cells. In addition, treatment of A2780cis cells with 5-aza-2′-deoxycytidine, a DNA-demethylating agent, restored GS expression and reduced CDDP resistance. In contrast, GS knockdown in CDDP-sensitive A2780 cells induced CDDP resistance.

**Conclusions:**

The results indicate that upregulation of GSH synthesis from glutamine via DNA methylation-mediated silencing of GS causes CDDP resistance in A2780cis cells. Therefore, glutamine metabolism could be a novel therapeutic target against CDDP resistance.

**Supplementary Information:**

The online version contains supplementary material available at 10.1186/s12885-021-07879-5.

## Background

Cisplatin (CDDP), a platinum-based drug, has been a mainstay of treatment in various cancers since it was approved by the U.S. Food and Drug Administration (FDA) in 1978 [1–3]. To date, CDDP remains commonly used as a first-line treatment for ovarian cancer in many countries. CDDP binds to nuclear DNA, particularly to the nucleophilic N7 sites of purine bases, with high affinity, thereby activating the DNA damage response [4–6]. However, some cancer cells develop CDDP resistance over time, leading to recurrences in up to 75% of patients with ovarian cancer [7–9]. CDDP-resistant cells often express elevated levels of glutathione (GSH) [10]. Research has shown that the levels of GSH in cancer cells are much higher than those in CDDP-treated cells [11–13]. GSH has high affinity for CDDP and competitively inhibits the binding of CDDP to DNA, causing CDDP resistance [10, 14]. Therefore, treatment with a GSH synthesis inhibitor can increase CDDP sensitivity [15].

Alterations in cellular metabolism are a crucial hallmark of cancer [16, 17], and cancer cells require both glutamine and glucose for their proliferation [18]. Glutamine contributes to the synthesis of not only nucleotides, amino acids, and proteins, but also of GSH, which is important for antioxidant defense [11]. Glutamine is the most abundant amino acid in serum, but it is often severely depleted in growing tumors due to nutrient-limited environments, and glutamine starvation may lead to rapid cancer cell death [11, 13, 19]. Extracellular glutamine is transported into cells and converted into glutamate by glutaminase (GLS). Glutamate is in turn used for α-ketoglutarate (α-KG) synthesis by glutamate dehydrogenase (GLUD). Conversely, glutamate is metabolized into glutamine by glutamine synthetase (GS), which is encoded by glutamate ammonia ligase (GLUL). The levels and functions of GS in tumors vary depending on the cellular context [20]. Low-invasive ovarian cancer cells express high levels of GS, whereas highly invasive ovarian cancer cells express low levels of GS [21]. Meanwhile, GS fuels nucleotide biosynthesis and facilitates growth of various cancer cells [14, 22–24].

Although recent research has revealed a relationship between CDDP resistance and glutamine metabolism, the exact mechanism is yet to be elucidated [25–29]. A preliminary hypothesis is that CDDP-resistant cells upregulate GSH production from glutamine, thereby attenuating CDDP-induced cytotoxicity. However, key factors that regulate the “resistance system” in cancer cells remain to be identified. Metabolome analysis is one of the powerful approaches to understanding the molecular mechanisms by which cancer cells acquire malignant potential [30].

This study aimed to determine the mechanisms by which ovarian cancer cells acquire CDDP resistance. Towards this goal, we conducted capillary electrophoresis-time-of-flight mass spectrometry (CE-TOFMS) [31, 32] to quantify the central carbon metabolites and amino acids in the human ovarian epithelial cancer cell line A2780 and the CDDP-resistant daughter cell line A2780cis and then performed glutamine metabolic flux analysis. Considering the higher levels of glutamine and GSH in CDDP-resistant cells, we hypothesized that reprogramming of glutamine metabolism contributes to CDDP resistance in cancer cells.

## Methods

### Materials

Cisplatin (Wako), 3-(4,5-dimethylthiazol-2-yl)-2,5-diphenyltetrazolium bromide (MTT) (Sigma-Aldrich), and Compound 968 (Merck Millipore) were dissolved in phosphate-buffered saline (PBS) and filtered through a 0.22-μm filter. 5-aza-2′-deoxycytidine (5-aza-dC; Tokyo chemical industry) was initially dissolved in dimethyl sulfoxide (DMSO) and further diluted with culture medium.

### Cell culture

The human ovarian cancer cell line A2780 (catalog no. 93112519) and the CDDP-resistant cell line A2780cis (catalog no. 93112517) were purchased from European Collection of Cell Cultures (ECACC) General Cell Collection in 2013. Cell lines were shown to be mycoplasma free using the Mycoalert kit from Lonza. A2780 and A2780cis cells [33, 34] were maintained in Roswell Park Memorial Institute (RPMI) 1640 medium (Sigma-Aldrich, Co. R8758) supplemented with 10% heat-inactivated fetal bovine serum (Equitech-bio) and an antibiotic-antimycotic mixed solution (Nacalai Tesque, Inc.). A2780cis cells were maintained in the presence of 1 μM CDDP to maintain CDDP resistance and cultured in the absence of CDDP for 24 h prior to each experiment. For glutamine starvation conditions, RPMI 1640 medium (Sigma-Aldrich, Co. R0883) were used. All cells were grown at 37 °C with 5% CO_2_.

### MTT assay

Cell viability was assessed using the MTT assay as follows. The cells were seeded in 96-well microtiter plates (4 × 10^3^ cells per well) and cultured for 24 h. For exposure to CDDP and compound 968, cells were cultured for an additional 48 h. For cell counting, 20 μL of MTT solution (5 mg/mL) was added to the culture medium, and cells were further cultured for 3 h to generate formazan crystals that were dissolved in 100 μL of DMSO after the culture medium had been removed. Viability was calculated from the absorbance of MTT formazan at 570 nm with a background correction of 690 nm using a TECAN microplate reader with Magellan software (Männedorf). The IC_50_ of CDDP after 48 h was calculated based on the viability curve.

### Metabolite extraction and standards

Cells were seeded in 6-well plates (2 × 10^5^ cells in 2 mL of medium) and cultured in regular medium with CDDP or low-glutamine medium for 48 h or the indicated time periods. For flux analysis, 1, 3, 6, and 12 h before sampling, the medium was replaced with medium containing ^13^C-labeled glutamine.

Sampling was performed by washing the cells twice with 5% mannitol solution, covering with 600 μL of methanol containing 25 μM internal standards (L-methionine sulfone, 2-(N-morpholino)-ethanesulfonic acid, and D-camphor-10-sulfonic acid), and homogenizing for 10 min to inactivate cellular enzymes. The cell-ethanol mixture was collected and mixed with Milli-Q water and chloroform in a 2:1:2 ratio. The resulting solutions were then centrifuged at 10,000 *g* for 3 min. The aqueous layers were collected for centrifugal filtration though 5-kDa cutoff filters (Merck Millipore) at 9100 *g* for 3 h. The extracted metabolites were concentrated using a centrifugal concentrator. The concentrated metabolites were dissolved in 25 μL of Milli-Q water containing 200 μM of the reference compounds (3-aminopyrrolidine and trimesate).

All metabolite standards were dissolved in Milli-Q water, 0.1 N HCl, or 0.1 N NaOH to obtain 10 mM or 100 mM stock solutions. Working standard mixtures were prepared by diluting stock solutions with Milli-Q water prior to injection into the CE-TOFMS. All chemicals used were of analytical or reagent grade.

### CE-TOFMS conditions for cationic and anionic metabolite analyses

The following instrumentation and measurement conditions were used for CE-TOFMS (Agilent Technologies, Santa Clara, CA, USA) as previously reported [30–32]. Briefly, for analyzing cations, a fused silica capillary (50 μm i.d. × 100 cm total length) was used with 1 M formic acid as the electrolyte [31]. Each sample was injected by applying a pressure of 50 mbar for 3 s and a continuous voltage of + 30 kV. A solution of 5 mM ammonium acetate and 0.5 μM reserpine in 50% (v/v) methanol in water was used as the sheath liquid at a flow rate of 10 μL/min. ESI-TOFMS was performed in the positive ion mode, and the capillary voltage was set to 4 kV. Automatic recalibration of each acquired spectrum was achieved using the masses of the reference standards ([^13^C isotopic ion of a protonated methanol dimer (2 MeOH+H)]+, m/z 66.0631) and ([hexakis (2,2-difluoroethoxy) phosphazene +H]+, m/z 622.0290). For analyzing anions, a commercially available COSMO (+) (chemically coated with cationic polymer) capillary (50 μm i.d., 5 cm total length) (Nacalai Tesque, Kyoto, Japan) was used with a 50 mM ammonium acetate solution (pH 8.5) as the electrolyte [32]. Each sample was injected by applying a pressure of 50 mbar for 30 s and a continuous voltage of − 30 kV. Methanol/5 mM ammonium acetate (50% v/v) containing 0.1 μM hexakis (2,2-difluoroethoxy) phosphazene was delivered as the sheath liquid at 10 μL/min. ESI-TOFMS was performed in the negative ion mode, and the capillary voltage was set to 3.5 kV. Automatic recalibration of each acquired spectrum was achieved using the masses of the reference standards (^13^C isotopic ion of deprotonated deuterated acetic acid dimer (2CD3COOH-H)- m/z 126.076001, Hexakis (2,2-difluoroethoxy) phosphazene +deprotonated deuterated acetic acid (M + CD3COOH-H)- m/z 683.054372). The other conditions were identical to those described previously.

### Metabolome data processing

Metabolome data were preprocessed with MasterHands ver.2 [35]. The peaks were identified by matching the m/z values and normalized migration times of corresponding external stand compounds. All of the identified peaks were changed manually, and noise-derived peaks were removed based on S/N values. All peak areas were normalized using internal standards, and the concentrations of each compound were calculated according to the relative area of the external standard compound. The average amount of each metabolite per cell was evaluated based on the number of viable cells in each cell line. The number of cells was determined using a hemocytometer.

Hierarchical clustering of metabolite levels for heat-map visualization was performed in MultiExperiment Viewer (MeV) [36].

### Western blot analysis

The protein levels of glutamine-related enzymes were determined via Western blot analysis. Briefly, cells were collected using cell scraper, washed once with PBS, and centrifuged at 5000 rpm for 1 min. Protein extracts were prepared by lysing cells in RIPA Buffer (Nacalai tesque) on ice for 10 min. Protein quantification was performed using a Broadford protein assay kit (BIO-RAD). After determining the protein concentration, protein samples were mixed with 5× loading buffer then boiled for 10 min at 96 °C. Samples (20 μg of protein) were separated using SDS-PAGE (7.5% gel) and then transferred to a polyvinylidene difluoride (PVDF) membrane with a Trans-Blot Turbo Transfer System (BIO-RAD).

The membrane was first blocked with PBST containing 4% BSA for 10 min at room temperature. Next, they were incubated with primary antibodies at 4 °C overnight and then incubated with secondary antibodies at room temperature for 2 h. The immunoreactive proteins on the membrane were analyzed using ECL detection reagents and Image Quant LAS 4000 (GE Healthcare). The antibodies used were as follows: anti-GLUL, 1:1000 (HPA007316, Atlas Antibodies); anti-GLS, 1:2000 (ab156876, Abcam); anti-β-actin, 1:10,000 (ab8226, Abcam); anti-rabbit IgG HRP-linked antibody, 1:10,000 (Cell Signaling); anti-mouse IgG HRP-linked antibody, 1:10,000 (Cell Signaling).

### Knockdown of GS expression

siRNA targeting human GS and negative control siRNA were purchased from Sigma-Aldrich. A2780 cells were separately seeded in 6-well culture plates at a density of 4 × 10^3^ cells/well and cultured for 24 h. For siRNA transfection, complexes of siRNA duplex and Lipofectamine RNAiMAX (Invitrogen) were formed in serum-free medium and added to the culture medium at a final concentration of 25 nM siRNA. For RNA extraction, transfected cells were harvested 48 h after transfection. The siRNA sequences for GS were as follows: 5′-GAUUGGACCUUGUGAAGGAdTdT-3′; 5′-UCCUUCACAAGGUCCAAUCdTdT-3′.

### Quantitative real-time polymerase chain reaction (qRT-PCR)

RNA was isolated from cells using RNeasy Mini kit (Qiagen) following the manufacturer’s instructions. RNA concentration was qualitatively assessed and quantified using NanoDrop 2000 (Thermo scientific). Total RNA (2 μg) was reverse transcribed to cDNA with a ReverTra Ace qPCR RT Master Mix (TOYOBO). RT-PCR was performed with SYBR Green RT-PCR Master Mix (TaKaRa) on a StepOnePlus Real-Time PCR System (Thermo Scientific). PCR cycles consisted of initial denaturation at 95 °C for 30 s, followed by 40 cycles of 95 °C for 30 s, 95 °C for 5 s, and 60 °C for 30 s. The relative expression of mRNA was calculated using the 2^−ΔΔCt^ method. Data were normalized to the expression of β-actin or RPL27. The sequences of primers used are listed in Table S1.

### DNA demethylation

For DNA demethylation, cells were seeded in 6-well plates at a density of 2 × 10^5^ cells per well). After overnight culture, 2 μM 5-aza-2′-deoxycytidine (5-aza-dC), a DNA methyltransferase inhibitor, was added to the culture medium, and cells were incubated for an additional 72 h. GS expression was determined using RT-PCR.

## Results

### Components of glutamine metabolism is increased in CDDP-resistant cells

**Fig. 1:**
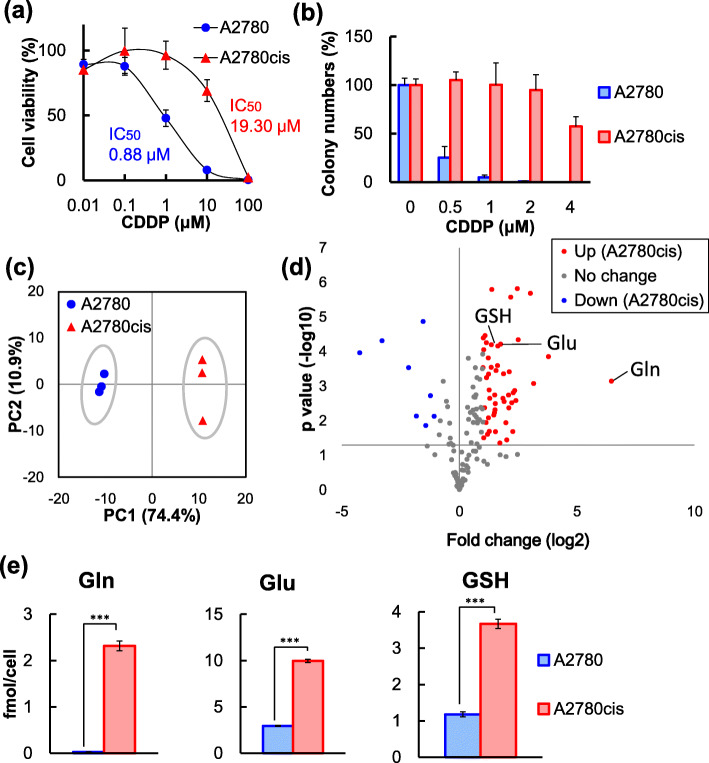
Increase in CDDP resistance and global metabolic changes in A2780cis cells. **a** Effects of CDDP treatment on the viability of A2780 and A2780cis cells. Cell viability was measured at 48 h after treatment using the MTT assay. **b** For colony formation assays, A2780 and A2780cis cells were cultured in the presence of CDDP (concentrations as indicated). Colonies were counted 9 days after plating. **c** Score plots of principal component analysis (PCA) of 189 intracellular metabolite levels in A2780 and A2780cis cells measured using CE-TOFMS. The contribution rate of PC1 and PC2 were 74.4 and 10.9%, respectively. **d** Volcano plots with the fold change of each metabolite and *p* values calculated using the Student’s t-test (*p* < 0.05). The averages metabolite levels in A2780cis cells were compared with those in A2780 cells (*n* = 3). Red dots depict significantly increased metabolites in A2780cis cells. Blue dots depict significantly decreased metabolites in A2780cis cells. Gray dots depict metabolites without significant differences. See also Table S2. **e** Levels of glutamine (Gln), glutamate (Glu), and glutathione (GSH) in A2780 and A2780cis cells. Data are shown as the mean ± SD of the three independent experiments. Statistical significance was determined using the Student’s t-test (**p < 0.01, ***p < 0.001)

Drug resistance is one of the most crucial challenges in cancer treatment. We used the CDDP-sensitive human ovarian cancer cell line A2780 and the CDDP-resistant cell line A2780cis, which was obtained by long-term exposure of A2780 cells to increasing concentrations of CDDP [33]. The MTT assay showed that the half-maximal inhibitory concentrations (IC_50_) for CDDP in A2780cis cells were approximately 20 times greater than those in A2780 cells (Fig. 1a). The colony formation capability of A2780cis cells was also greater than those of A2780 cells (Fig. 1b).

These cell lines were also evaluated using CE-TOFMS to determine the metabolic pathways responsible for CDDP resistance. The 189 metabolites in the major energy metabolism pathway were identified in extracts of A2780 and A2780cis cells using authentic standards. Principal component analysis (PCA) of metabolites revealed global metabolic changes between A2780 and A2780cis cells (Fig. 1c). The score plots along with the first principal component axis (PC1) showed marked differences between these cell lines. As shown by the volcano plots, the levels of 50 metabolites were increased to 2-fold or more, whereas those of 8 metabolites were decreased to 0.5-fold or less in A2780cis cells compared with those in A2780 cells (Fig. 1d and Table S2). The most remarkably increased metabolite in A2780cis cells was glutamine, the levels of which were 88-fold higher than those in A2780 cells (Fig. 1e). In addition, the levels of glutamate and glutathione, which are synthesized from glutamine, were also significantly increased in A2780cis cells (Fig. 1e). This shows that in A2780cis cells, CDDP resistance was elevated with metabolic changes, including increases in the components of glutamine metabolism.

### Reprogramming of glutamine metabolism enhances CDDP resistance in ovarian cancer cells

Glutamine as one of the main energy sources is involved in cancer cell proliferation, inhibition of apoptosis, and cell signaling [19, 37, 38]. Glutamine is converted to glutamate, which is a metabolic intermediate channeled into the tricarboxylic acid (TCA) cycle and GSH synthesis [39]. Taken together with our observations that levels of glutamine and GSH are higher in CDDP-resistant cells, we hypothesized that reprogramming of glutamine metabolism contributes to CDDP resistance in cancer cells. To test our hypothesis, we conducted three experiments.

**Fig. 2:**
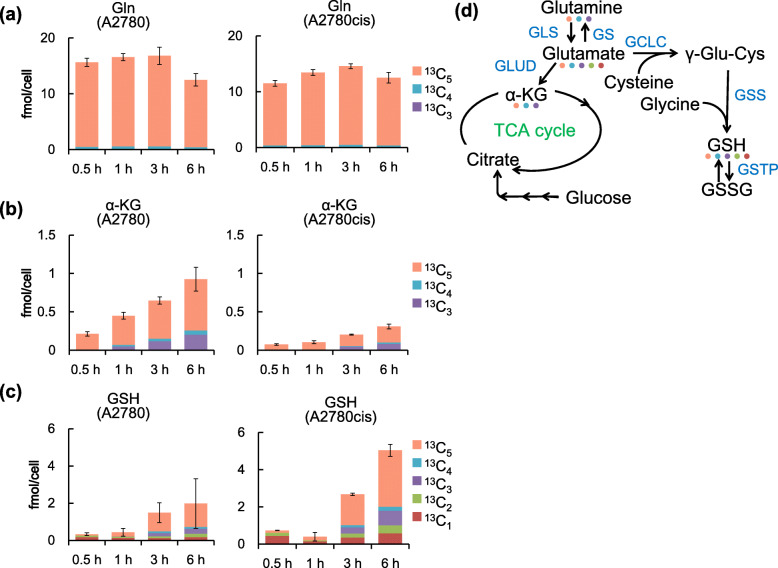
Metabolic flux analysis using isotopically labelled glutamine in A2780 and A2780cis cells. **a**, **b**, and **c** Isotopologue distribution of metabolites in A2780 and A2780cis cells. Cells were incubated with medium containing glutamine isotopically labeled at all five carbon atoms (^13^C_5_-glutamine) for the indicated time periods. Carbon fluxes from glutamine to Glu, GSH, and α-ketoglutarate (α-KG) were determined using CE-TOFMS. Each bar color corresponds to the number of ^13^C replaced with ^12^C in the metabolites. Data are shown as the mean ± SD of three independent experiments. **d** A pathway map of glutamine metabolism. Metabolites and catalytic enzymes are shown in black and blue, respectively. The colored dots show the ^13^C isotopically labeled metabolites, and the color corresponds to the icons in Fig. 2a–c on the left

First, to examine the difference in glutamine metabolism between A2780 and A2780cis cells via metabolic flux analysis using glutamine isotopically labeled at all five carbon atoms (^13^C_5_-glutamine). For this analysis, we cultured these cell lines in medium containing labeled glutamine and determined the levels of metabolites produced from labeled glutamine using CE-TOFMS. As expected, the levels of ^13^C_5_-labeled glutamine were similar in both cell lines, suggesting that glutamine incorporation was not changed in A2780 and A2780cis cells (Fig. 2a, orange). Meanwhile, the levels of labeled TCA cycle metabolites (^13^C_1_–^13^C_5_), including α-KG, were lower in A2780cis cells than in A2780 cells. (Fig. 2b, and Fig. S1). In contrast, labeled GSH was actively produced from labeled glutamine in A2780cis cells (Fig. 2c). These results suggest that glutamine is preferentially involved in GSH production in CDDP-resistant cells.

**Fig. 3:**
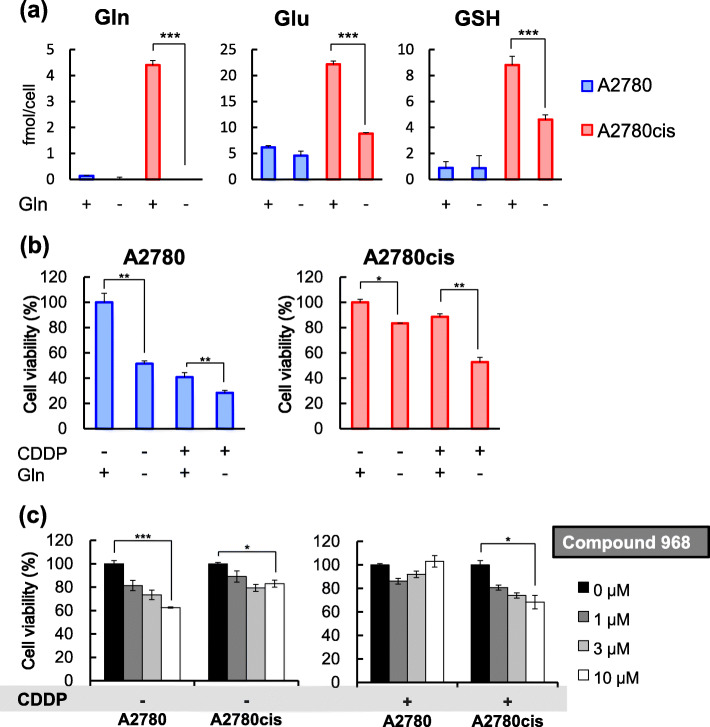
Glutamine starvation reduces the GSH level and CDDP resistance in A2780cis cells. **a** Levels of Gln, Glu, and GSH in A2780 and A2780cis cells cultured in the presence (+) or absence (−) of glutamine. **b** Effects of glutamine starvation on CDDP resistance. A2780 and A2780cis cells were treated with 3 μM CDDP in the presence (+) or absence (−) of glutamine for 48 h. **c** A2780 and A2780cis cells were cultured for 48 h in medium containing CDDP (0 or 10 μM) and the GLS inhibitor compound 968 (0, 1, 3, or 10 μM). Cell viability was measured using the MTT assay. Data are shown as the mean ± SD of the three independent experiments. The differences were analyzed by using the Student’s *t*-test (*p < 0.05, **p < 0.01, ***p < 0.001)

Second, we used CE-TOFMS to analyze the metabolic profiles of A2780 and A2780cis cells cultured under glutamine starvation conditions. In agreement with the results of the first experiment, the levels of glutamate and GSH in A2780cis cells were significantly decreased under glutamine starvation conditions, while those in A2780 cells were not affected by glutamine starvation (Fig. 3a). We also found that levels of various metabolites were changed by glutamine starvation in both cell lines (Table S3, S4, S5, S6). These results indicate that glutamine starvation causes metabolic reprogramming.

Third, we assessed CDDP resistance of A2780 and A2780cis cells in the presence or absence of glutamine. The viability of A2780 cells was decreased not only by CDDP treatment alone, but also by glutamine starvation alone, suggesting that A2780 cells depend on glutamine to sustain their proliferation (Fig. 3b left). In contrast, the viability of A2780cis cells was neither drastically affected by CDDP treatment alone nor glutamine starvation alone (Fig. 3b right). Importantly, however, glutamine starvation reduced the viability of A2780cis cells in the presence of CDDP. The effect of glutamine starvation on the viability of A2780cis cells was also greater than those of A2780 cells in the presence of CDDP. These results indicate that A2780cis cells depend on glutamine to induce CDDP resistance.

The results of these three experiments collectively support the hypothesis that glutamine is utilized preferentially for GSH production rather than for TCA cycle metabolite production in A2780cis cells. This reprogramming of glutamine metabolism enhances CDDP resistance.

The result that glutamine metabolism plays an important role in CDDP resistance prompted us to examine the effect of treatment with a glutamine metabolism inhibitor on CDDP resistance. Accordingly, we treated cells with a constant concentration of CDDP (10 μM) and various concentrations of compound 968, a GLS inhibitor, and analyzed cell viability. Consistent with the results presented in Fig. 3b, the viability of A2780cis cells in the presence of CDDP was decreased by compound 968 treatment in a concentration-dependent manner (Fig. 3c). These results show that treatment with a GLS inhibitor enhances the cytotoxic effects of CDDP on CDDP-resistant cells.

### CDDP resistance in A2780cis cells is caused by DNA methylation-mediated silencing of GS expression

**Fig. 4:**
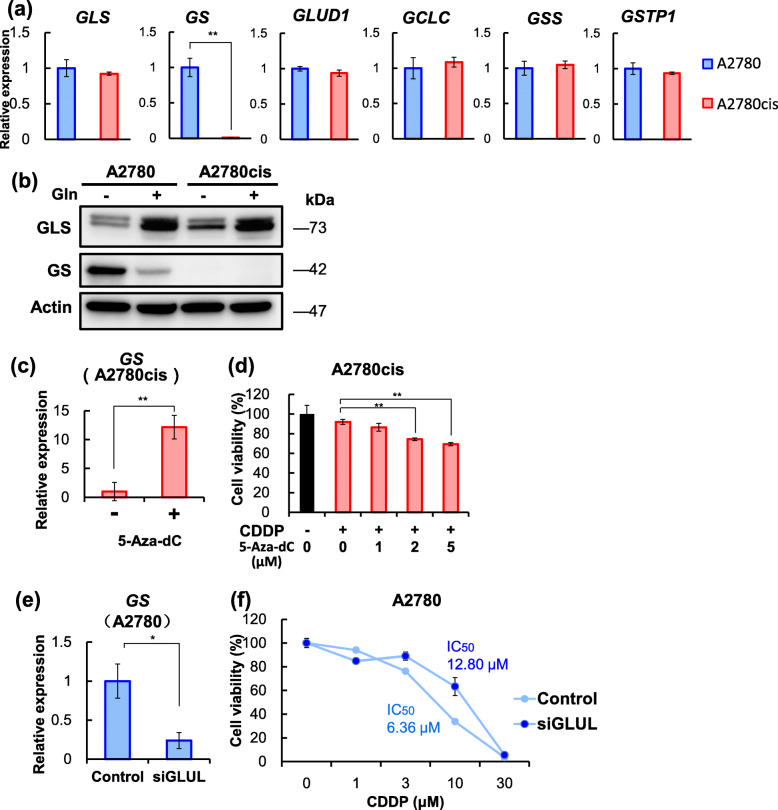
CDDP resistance in A2780cis cells is caused by DNA methylation-medicated silencing of GS expression. **a** RT-PCR analysis of glutamine metabolism enzymes. **b** Western blotting of glutamine metabolism enzymes. “+“indicates culture medium with normal glutamine concentration, and “−“indicates culture medium without glutamine. Cells were cultured in the absence or presence of glutamine for 48 h. **c** RT-PCR analysis of GS expression in A2780cis cells. Cells were cultured for 72 h in the absence or presence of 5-Aza-dC (2 μM). **d** Effects of 5-Aza-dC on CDDP-induced cytotoxicity. A2780cis cells were cultured for 72 h in medium containing CDDP (0 or 10 μM) and 5-Aza-dC (0, 1, 2, or 5 μM). Cell viability was measured using the MTT assay. **e** Confirmation of GS knockdown in A2780 cells using RT-PCR analysis. **f** Effects of CDDP knockdown on CDDP-induced cytotoxicity. A2780 cells transfected with control siRNA or GS siRNA were cultured for 72 h in the presence of CDDP (concentrations as indicated). Cell viability was measured using the MTT assay. Data are shown as the mean ± SD of the three independent experiments. The differences were analyzed using the Student’s *t*-test or one-way ANOVA with Dunnett’s multiple comparison (*p < 0.05, **p < 0.01)

As shown in Fig. 3b and c, CDDP-induced cytotoxicity against A2780cis cells was enhanced by glutamine starvation or GLS inhibitor treatment. Therefore, we next examined the expression of glutamine metabolism enzymes. Unexpectedly, real-time polymerase chain reaction (RT-PCR) and Western blot analyses showed that there were no significant differences in the levels of GLS between A2780 cells and A2780cis cells (Fig. 4a). Meanwhile, GLS expression was induced in the presence of glutamine, a substrate for GLS, in both cell lines (Fig. 4b). In addition, both cell lines expressed similar levels of GLUD1, GCLC, GSS, and GSTP1, a major drug-metabolizing enzyme [40, 41] (Fig. 4a). We also performed western blotting to examine the expression level of GS in medium with normal glutamine concentration and medium without glutamine.

Interestingly, we found that GS expression was almost completely suppressed in A2780cis cells (Fig. 4a), whereas A2780 cells expressed a detectable level of GS in the presence of glutamine and a higher level of GS in the absence of glutamine (Fig. 4b). Thus, it is believed that the absence of glutamine causes cells to express a higher level of GS, which supplements glutamine level in the cells. However, GS expression did not increase in A2780cis cells even in the absence of glutamine. Bott et al. recently reported that the GS promoter is methylated in human mammary epithelial cells and that GS expression is induced by Myc-mediated promoter demethylation [23]. Therefore, to determine whether suppression of GS expression in A2780cis cells is due to DNA methylation, we treated A2780cis cells with 5-aza-2′-deoxycytidine (5-Aza-dC), an inhibitor of DNA methyltransferases, and analyzed GS expression using RT-PCR. As expected, GS expression was markedly increased by 5-Aza-dC treatment, indicating that GS expression is suppressed by DNA methylation (Fig. 4c). We further investigated whether 5-Aza-dC treatment attenuates CDDP resistance in A2780cis cells. We treated A2780cis cells with a constant concentration of CDDP (10 μM) and various concentrations of 5-Aza-dC and found promising findings. The viability of A2780cis cells in the presence of CDDP was decreased by 5-Aza-dC treatment in a concentration-dependent manner (Fig. 4d). This result demonstrates that CDDP resistance in A2780cis cells is diminished by 5-Aza-dC treatment.

Finally, to clarify the role of GS in CDDP resistance, we knocked down GS expression in parental A2780 cells, which express GS and are CDDP sensitive, and evaluated cell viability in the presence of various concentrations of CDDP (Fig. 4e). Consistent with the results that CDDP-resistant A2780cis cells scarcely expressed GS, GS knockdown in A2780 cells caused an approximately two-fold increase in the IC_50_ value for CDDP (Fig. 4f). Collectively, these results indicate that CDDP resistance in A2780cis cells is induced, at least in part, by DNA methylation-mediated silencing of GS expression.

## Discussion

Through catalyzing the formation of glutamine from glutamate and ammonia, GS functions in various processes in cancer cells, including nucleotide biosynthesis, cell proliferation [14, 22, 24], and cell invasion [42]. However, the roles of GS in CDDP resistance in cancer cells have not been elucidated. In this study, we found global metabolic changes in CDDP-resistant ovarian cancer cells. First, levels of glutamine, glutamate, and GSH, which is associated with drug resistance, were higher in A2780cis cells than those in A2780 cells (Fig. 1e). Second, levels of TCA cycle metabolites synthesized from glutamine were lower in A2780cis cells than those in A2780 cells (Fig. 2b and Fig. S1). To our best knowledge, this is the first study to report the importance of glutamine metabolic reprogramming in CDDP resistance.

In addition, we found that glutamine starvation reduced the levels of glutamine, glutamate, and GSH and, accordingly, CDDP resistance in A2780cis cells (Fig. 3a and b). Treatment of A2780cis cells with compound 968, a GLS inhibitor, also diminished CDDP resistance (Fig. 3c). Furthermore, treatment of A2780cis cells with 5-Aza-dC restored the expression of GS and reduced CDDP resistance (Fig. 4c and d). In summary, glutamine starvation, GLS inhibition, and 5-Aza-dC treatment reduced CDDP resistance in A2780cis cells. These results indicate that GSH production from glutamine plays a crucial role in the development of CDDP resistance. Consistent with these observations, GS knockdown in CDDP-sensitive A2780 cells induced CDDP resistance (Fig. 4f).

**Fig. 5:**
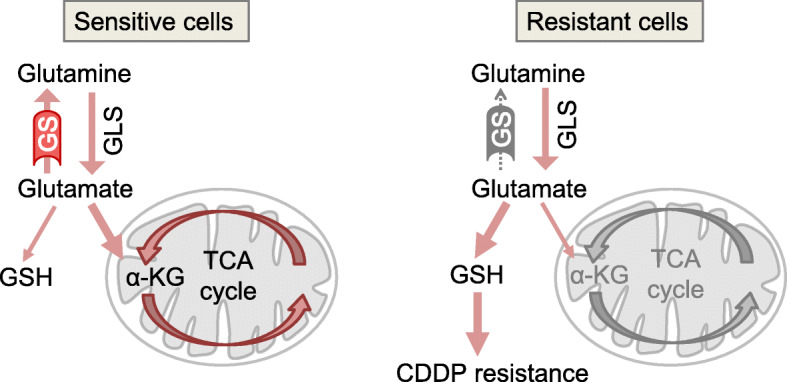
A model for CDDP resistance development via reprogramming of glutamine metabolism in ovarian cancer cells. In CDDP-sensitive cancer cells, both GLS and GS are expressed, and low levels of GSH are produced from glutamate. In contrast, in CDDP-resistant cells, GS expression is suppressed by DNA methylation, whereas GLS expression is maintained, and thereby high levels of GSH are produced. This reprogramming of glutamine metabolism causes CDDP resistance

Based on our results, we proposed a hypothesis for the development of CDDP resistance in ovarian cancer cells (Fig. 5). In CDDP-sensitive cells, both GLS and GS are expressed, and low levels of GSH are produced from glutamate. In contrast, in CDDP-resistant cells, GS expression is suppressed by DNA methylation, while GLS expression is maintained. Thus, high levels of GSH are produced, and levels of TCA cycle metabolites synthesized from glutamine are decreased. This reprogramming of glutamine metabolism causes CDDP resistance. The mechanisms and functional roles of decreases in levels of glutamine-derived TCA cycle metabolites remain to be elucidated in CDDP-resistant cells. However, we speculate that in addition to GS, other genes may also be silenced by DNA methylation. These alterations in gene expression may contribute to a metabolic shift from TCA cycle metabolite synthesis to GSH synthesis. This reduction of TCA cycle activity might cause cell growth suppression, a decrease in CDDP-induced DNA damage, and CDDP resistance.

Our present study shows that GS expression is almost completely suppressed via DNA methylation in CDDP-resistant A2780cis cells (Fig. 4a, b, and c). GS knockdown in CDDP-sensitive A2780 cells induced CDDP resistance (Fig. 4f). Interestingly, Yang et al. reported that low-invasive ovarian cancer cells express high levels of GS, whereas highly invasive ovarian cancer cells express low levels of GS [42]. Other studies reported that daunorubicin-resistant acute lymphoblastic leukemia cells lack GS expression [43]. In addition, GS knockdown in non-small cell lung cancer and hepatocellular carcinoma cells enhances resistance to gefitinib and sorafenib, respectively [44, 45]. GS knockout in non-small-cell lung carcinoma cells also increases resistance to pazopanib and docetaxel [46]. Thus, we infer that GS inactivation is a crucial step in acquiring malignant potential, including drug resistance, in various cancers.

## Conclusions

Our results highlight the importance of glutamine metabolism in CDDP resistance in ovarian cancer cells. We found that levels of glutamine, glutamate, and GSH in A2780cis cells were significantly higher than those in A2780 cells. GS expression was almost completely suppressed in A2780cis cells. In addition, treatment of A2780cis cells with 5-aza-dC restored GS expression and reduced CDDP resistance. Thus, targeting glutamine metabolism, particularly with DNA methyltransferase inhibitors, could be a promising strategy to overcome chemotherapy resistance in various cancers.

## Supplementary Information

**Additional file 1.**

## Data Availability

The datasets used and/or analyzed during the current study are available from the corresponding author on reasonable request.

## Abbreviations

5-aza-dC: 5-Aza-2′-deoxycytidine
CDDP: Cis-diaminedichloroplatinum
CE-TOFMS: Capillary electrophoresis–time-of-flight mass spectrometry
GLS: Glutaminase
GSH: Glutathione
GS: Glutamine synthetase
MTT: 3-(4,5-Dimethylthiazol-2-yl)-2,5-diphenyltetrazolium bromide
RT-PCR: Real-time polymerase chain reaction
TCA: Tricarboxylic acid
