# Supplementary material for: Reprogramming of glutamine metabolism via glutamine synthetase silencing induces cisplatin resistance in A2780 ovarian cancer cells
Source: BMC Cancer. 2021 Feb 17;21:174. doi: 10.1186/s12885-021-07879-5 (PMC7891143; doi:10.1186/s12885-021-07879-5)
Supplement: Supplementary file 1 — Additional file 1. [file 12885_2021_7879_MOESM1_ESM.pdf]

Supplementary Files for

Reprogramming of glutamine metabolism via glutamine synthetase silencing induces cisplatin resistance in A2780 ovarian cancer cells

Jing Guo, Kiyotoshi Satoh*, Sho Tabata, Masaru Mori, Masaru Tomita, and Tomoyoshi Soga

*Correspondence to Kiyotoshi Satoh, E-mail: ksatoh@ttck.keio.ac.jp

This file includes:
4 Figures and 6 Tables

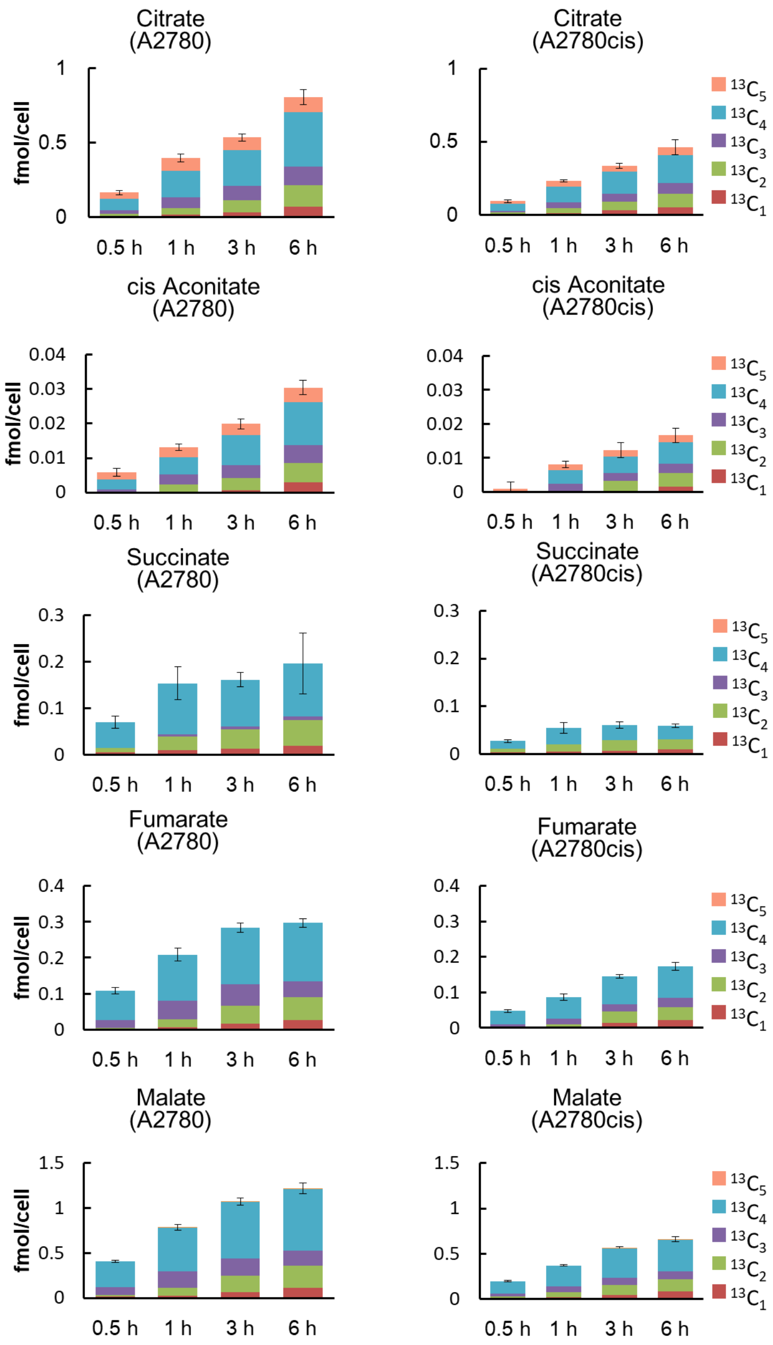

Figure S1. Metabolic flux analysis using isotopically labelled glutamine in A2780 and A2780cis cells.

Isotopologue distribution of metabolites in A2780 and A2780cis cells. Cells were incubation with medium containing glutamine isotopically labeled at all five carbon atoms ($^{13}C_5$-glutamine) for the indicated time periods. Carbon fluxes from glutamine to TCA cycle metabolites were determined using CE-TOFMS. Each bar color corresponds to the number of ^{13}C replaced with ^{12}C in the metabolites. Data are shown as the mean ± SD of the three independent experiments. See also Figure 2.

Table S1. Primer sequences used for RT-PCR analysis.

GS	Forward：	5‘ AGGGTTAAAGAGGGCAACCC 3’
	Reverse：	5’ GAGGTGGTCATGGTGGAAGG 3’
GLUD1	Forward：	5‘GGGAGGTCATCGAAGGCTAC 3’
	Reverse：	5’ TCACATCAGTGCTGTAACGGA 3’
GCLC	Forward：	5’ GTTCTCAAGTGGGGCGATGA 3’
	Reverse：	5’ TTCTCCCCAGACAGGACCAA 3’
GSS	Forward：	5’ GAACCGTTCGCGGAGGAAA 3’
	Reverse：	5’ TATCCTGCAAGAGGCTCCCC 3’
GSTP1	Forward：	5’ GAGGACCTCCGCTGCAAATA 3’
	Reverse：	5’ CAGCAGGGTCTCAAAAGGCT 3’
β-actin	Forward：	5’ CCAGCCTTCCTTCCTGGGCATGG 3’
	Reverse：	5’ TTGGCGTACAGGTCTTTGCGGAT 3’
GLS	Forward：	5’ TAGCTTGGAAGATTTGCTGT 3’
	Reverse：	5’ CCTGTAGATTTGAGTGCTGT 3’
RPL27	Forward：	5’ CTGTCGTCAATAAGGATGTCT 3’
	Reverse：	5’ CTTGTTCTTGCCTGTCTTGT 3’

Table S2. Metabolites with significant differences in expression between A2780 and A2780cis cells

The levels of 189 metabolites in A2780 and A2780cis cells in the presence of glutamine were determined via CE-TOFMS using 513 metabolite standards. The levels of 50 metabolites in A2780cis cells were at least 2.0-fold higher than those in A2780 cells, whereas those of 8 metabolites were lower by 0.5-fold or less than those in A2780 cells (n=3). Statistical significance was determined using the Students' *t* test ($p<0.05$). See also Figure 1C.

	Fold (A2780cis/A2780)	Average (fmol/cell) (A2780)	Average (fmol/cell) (A2780cis)	p valve	
Gln	88.62	0.026	2.317	7.20E-04	Up
Glycerophosphorylcholine	13.82	0.208	2.871	1.41E-04	Up
Urea	8.92	0.447	3.993	8.41E-04	Up
Asp	8.12	0.145	1.179	2.08E-06	Up
o-Acetylcarnitine	5.69	0.233	1.328	4.56E-05	Up
Carnitine	5.52	0.007	0.038	1.52E-06	Up
Choline	5.31	0.010	0.055	2.62E-03	Up
Glycerophosphate	5.12	0.126	0.644	1.34E-03	Up
Ala	4.91	1.384	6.794	1.56E-03	Up
4-Oxopentanoate	4.83	0.037	0.180	2.06E-02	Up
GABA	4.69	0.044	0.206	3.01E-03	Up
Hypotaurine	4.54	0.142	0.647	2.68E-06	Up
beta-Ala	4.36	0.107	0.467	3.86E-04	Up
Taurine	4.31	0.391	1.686	4.26E-03	Up
Kynurenine	4.27	0.007	0.030	1.83E-03	Up
Ophthalmate	4.05	0.001	0.003	3.59E-02	Up
Creatinine	3.94	0.016	0.064	1.14E-02	Up
G1P	3.75	0.032	0.120	2.79E-03	Up
alpha-Aminoadipate	3.68	0.005	0.019	4.43E-04	Up
3-Hydroxybutyrate	3.61	0.049	0.177	2.25E-03	Up
Glu	3.37	2.953	9.962	6.15E-05	Up
Glycolate	3.31	0.070	0.233	4.41E-02	Up
Glutathione (GSH)	3.11	1.179	3.667	6.97E-05	Up
gamma-Butyrobetaine	3.09	0.009	0.027	2.55E-04	Up
threo-beta-methylaspartate	2.95	8.221	24.259	3.54E-04	Up
GDP	2.90	0.004	0.012	2.07E-02	Up
Val	2.89	0.117	0.339	3.22E-03	Up
trans-Cinnamate	2.87	0.227	0.650	4.83E-03	Up
Azelate	2.84	0.009	0.026	1.81E-03	Up
Cysteine sulfinate	2.82	0.036	0.103	5.86E-03	Up
2-Hydroxybutyrate	2.77	0.016	0.045	6.85E-03	Up
Met	2.60	0.079	0.206	1.62E-06	Up
Pro	2.57	1.060	2.721	6.38E-05	Up

Nicotinamide	2.56	0.011	0.028	2.87E-04	Up
Ornithine	2.45	0.117	0.286	2.26E-03	Up
Carnosine	2.42	0.002	0.006	2.04E-02	Up
Carbamoyl phosphate	2.41	0.453	1.091	1.29E-03	Up
N-gamma-ethylglutamine	2.37	0.003	0.006	4.51E-04	Up
Thiamine	2.36	0.003	0.007	8.29E-03	Up
Betaine	2.35	0.045	0.105	1.53E-04	Up
CDP	2.29	0.006	0.013	2.46E-02	Up
Creatine	2.23	1.055	2.347	5.70E-04	Up
Leu	2.22	0.415	0.922	5.58E-05	Up
Syringate	2.20	0.077	0.169	1.13E-02	Up
Phe	2.13	0.094	0.201	3.49E-05	Up
2AB	2.11	0.021	0.045	4.23E-03	Up
Thr	2.06	0.484	0.996	8.89E-05	Up
NADP+	2.05	0.005	0.010	3.13E-02	Up
ADP	2.03	0.131	0.266	4.07E-05	Up
Lactate	2.01	11.655	23.389	2.92E-04	Up
N-Acetylputrescine	0.48	0.025	0.012	7.41E-03	Down
SAM+	0.43	0.038	0.016	1.90E-03	Down
N-Acetylglucosamine 6-phosphate	0.37	0.113	0.042	1.37E-02	Down
Argininosuccinate	0.34	0.043	0.015	1.36E-05	Down
Arg	0.28	0.819	0.231	7.33E-03	Down
Ser	0.22	0.675	0.151	2.91E-04	Down
Cystathionine	0.10	0.558	0.057	4.90E-05	Down
2-Hydroxyglutarate	0.05	0.127	0.007	1.09E-04	Down

Table S3. Metabolites with significant differences in expression between A2780 and A2780cis cells in the presence of glutamine

The levels of 77 metabolites in A2780 and A2780cis cells were determined via CE-TOFMS using 114 metabolite standards. The levels of 28 metabolites in A2780cis cells were at least 1.5-fold higher than those in A2780 cells, whereas those of 7 metabolites were lower by 0.67-fold or less than those in A2780 cells (n=3). Statistical significance was determined by Students' *t* test ($p<0.05$). See also Figure 3A.

	Fold (A2780cis/A2780)	Average (fmol/cell) (A2780)	Average (fmol/cell) (A2780cis)	p value	
Gln	31.67	0.139	4.400	5.26E-04	Up
Val	29.52	0.017	0.512	3.07E-05	Up
Ala	13.51	0.944	12.753	2.53E-04	Up
Asp	11.90	0.157	1.865	4.86E-04	Up
Met	11.67	0.028	0.322	4.94E-03	Up
Phe	10.40	0.029	0.303	1.84E-04	Up
F1,6P	10.23	0.052	0.530	1.33E-03	Up
Glutathione (GSH)	9.97	0.885	8.816	1.52E-04	Up
Lys	9.17	0.019	0.171	3.45E-03	Up
G6P	6.71	0.018	0.123	3.94E-03	Up
F6P	5.34	0.006	0.034	2.74E-02	Up
Glu	3.58	6.191	22.182	4.55E-05	Up
GABA	3.56	0.123	0.439	1.38E-05	Up
G1P	3.50	0.068	0.236	2.83E-03	Up
Cysteine sulfinate	3.28	0.041	0.135	2.86E-03	Up
Thr	2.97	0.535	1.589	7.54E-06	Up
CoA	2.85	0.005	0.015	1.83E-02	Up
Hypotaurine	2.56	0.666	1.705	6.26E-06	Up
Fumarate	2.55	0.073	0.185	9.49E-03	Up
ATP	2.32	1.826	4.240	4.88E-02	Up
Malate	2.30	0.307	0.707	5.18E-03	Up
DHAP	2.26	0.017	0.039	1.61E-02	Up
Pyruvate	2.18	0.346	0.755	5.88E-03	Up
Leu	2.12	0.731	1.546	3.49E-04	Up
Glycerophosphate	1.77	0.546	0.968	2.21E-02	Up
Taurine	1.67	0.440	0.737	4.37E-03	Up
Ile	1.66	0.985	1.632	2.46E-05	Up
PEP	1.55	0.021	0.033	1.40E-02	Up
Gly	0.58	17.570	10.164	2.18E-04	Down
Citrulline	0.55	0.127	0.070	2.44E-03	Down
Lactate	0.55	26.650	14.583	3.68E-04	Down
Ser	0.36	0.666	0.240	2.70E-04	Down
Cystathionine	0.24	0.606	0.145	3.38E-04	Down
Arg	0.20	4.333	0.882	1.65E-03	Down
2-Hydroxyglutarate	0.17	0.156	0.026	4.85E-02	Down

Table S4. Metabolites with significant differences in expression between A2780 and A2780cis cells under glutamine starvation

The levels of 63 metabolites in A2780 and A2780cis cells in the absence of glutamine were determined via CE-TOFMS using 114 metabolite standards. The levels of 10 metabolites in A2780cis cells were at least 1.5-fold higher than those in A2780 cells, whereas those of 8 metabolites were lower by 0.67-fold or less than those in A2780 cells (n=3). Statistical significance was determined by the Students' *t* test ($p<0.05$). See also Figure 3A.

	Fold (A2780cis/A2780)	Average (fmol/cell) (A2780)	Average (fmol/cell) (A2780cis)	p value	
Hypotaurine	9.78	0.220	2.151	1.38E-06	Up
Ala	7.93	0.417	3.306	3.64E-05	Up
Glutathione (GSH)	5.31	0.866	4.602	1.27E-02	Up
Creatine	4.98	4.520	22.521	3.13E-05	Up
Taurine	4.57	0.336	1.535	2.26E-03	Up
GABA	2.7	0.156	0.423	2.07E-04	Up
beta-Ala	2.58	0.200	0.517	2.61E-04	Up
Glycerophosphate	2.37	0.885	2.096	2.46E-02	Up
Glu	1.93	4.570	8.814	1.20E-02	Up
CDP	1.64	0.032	0.052	3.13E-02	Up
Citrulline	0.63	0.119	0.076	2.42E-02	Down
Gly	0.57	50.721	29.114	3.76E-02	Down
Tyr	0.55	1.677	0.928	4.62E-02	Down
S7P	0.34	0.164	0.056	2.74E-03	Down
Cystathionine	0.3	0.351	0.104	2.15E-02	Down
3PG	0.3	0.181	0.054	8.60E-03	Down
2-Hydroxyglutarate	0.27	0.211	0.058	3.67E-02	Down
Trp	0.23	0.362	0.083	1.73E-02	Down

Table S5. Metabolites significantly changed under glutamine starvation in A2780 cells

The levels of 70 metabolites in A2780 cells in the presence (Gln+) or absence (Gln-) of glutamine were determined via CE-TOFMS using 114 metabolite standards. The levels of 27 metabolites in the absence of glutamine were at least 1.5-fold higher than those in the presence of glutamine, whereas those of 5 metabolites were lower by 0.67-fold or less than those in the presence of glutamine (n=3). Statistical significance was determined using the Students' *t* test (p<0.05). See also Figure 3A.

	Fold (Gln-/Gln+)	Average (fmol/cell) (Gln+)	Average (fmol/cell) (Gln-)	p value	
Val	102.13	0.017	1.773	2.41E-02	Up
Lys	91.96	0.019	1.719	3.40E-02	Up
F1,6P	46.24	0.052	2.397	9.80E-03	Up
Phe	45.25	0.029	1.318	1.94E-02	Up
Met	38.60	0.028	1.064	1.77E-02	Up
DHAP	24.45	0.017	0.424	3.85E-02	Up
Ru5P	13.78	0.010	0.138	2.27E-02	Up
IMP	11.29	0.009	0.101	1.15E-02	Up
Thr	10.81	0.535	5.781	1.29E-02	Up
GTP	9.81	0.212	2.079	2.34E-02	Up
S7P	8.29	0.020	0.164	5.83E-03	Up
G1P	6.68	0.068	0.452	1.01E-03	Up
G6P	6.34	0.018	0.117	8.14E-04	Up
Ser	5.14	0.666	3.422	1.09E-02	Up
Leu	4.66	0.731	3.403	2.96E-02	Up
GDP	4.35	0.024	0.103	1.50E-02	Up
Carnosine	4.13	0.004	0.016	2.81E-02	Up
2PG	3.51	0.008	0.029	1.37E-03	Up
Tyr	3.31	0.507	1.677	2.25E-02	Up
3PG	3.13	0.058	0.181	2.00E-02	Up
PEP	2.89	0.021	0.062	1.77E-04	Up
Gly	2.89	17.570	50.721	1.75E-02	Up
dTTP	2.70	0.018	0.047	7.76E-03	Up
SAM+	2.28	0.019	0.044	1.05E-03	Up
ADP	2.27	0.257	0.585	3.77E-02	Up
AMP	2.23	0.053	0.119	9.58E-05	Up
Asn	2.21	6.985	15.414	2.96E-02	Up
Cystathionine	0.58	0.606	0.351	2.48E-02	Down
Taurine	0.56	0.440	0.247	3.13E-03	Down
Ala	0.44	0.944	0.417	2.36E-02	Down
Hypotaurine	0.33	0.666	0.220	2.36E-04	Down
beta-Ala	0.28	0.716	0.200	1.18E-04	Down

Table S6. Metabolites significantly changed under glutamine starvation in A2780cis cells

The levels of 63 metabolites in A2780cis cells in the presence (Gln+) or absence (Gln-) of glutamine were determined via CE-TOFMS using 114 metabolite standards. The levels of 23 metabolites in the absence of glutamine were at least 1.5-fold higher than those in the presence of glutamine, whereas those of 9 metabolites were lower by 0.67-fold or less than those in the presence of glutamine (n=3). Statistical significance was determined using the Students' *t* test ($p<0.05$). See also Figure 3A.

	Fold (Gln-/Gln+)	Average (fmol/cell) (Gln+)	Average (fmol/cell) (Gln-)	p value	
Ser	17.25	0.240	4.138	7.52E-04	Up
Creatine	5.32	4.233	22.521	8.09E-04	Up
Lys	3.87	0.171	0.663	1.08E-02	Up
F1,6P	3.81	0.530	2.021	1.96E-05	Up
CDP	3.01	0.017	0.052	1.67E-02	Up
Gly	2.86	10.164	29.114	9.24E-04	Up
Arg	2.65	0.882	2.336	6.13E-03	Up
GDP	2.64	0.051	0.135	2.48E-03	Up
Thr	2.58	1.589	4.103	5.16E-04	Up
Phe	2.33	0.303	0.705	1.00E-02	Up
AMP	2.30	0.057	0.130	9.65E-05	Up
Glycerophosphate	2.16	0.968	2.096	3.10E-02	Up
Val	2.15	0.512	1.103	8.71E-03	Up
Taurine	2.08	0.737	1.535	2.15E-02	Up
2-Hydroxyglutarate	2.05	0.026	0.054	9.45E-03	Up
ADP	2.02	0.366	0.741	5.52E-05	Up
Met	1.95	0.322	0.629	7.68E-04	Up
SAM+	1.74	0.024	0.043	1.04E-03	Up
Tyr	1.73	0.537	0.928	7.36E-03	Up
Glutathione (GSSG)	1.66	1.217	2.024	1.47E-02	Up
Asn	1.57	6.229	9.782	3.35E-03	Up
CTP	1.57	0.296	0.465	1.92E-02	Up
G1P	1.56	0.236	0.369	8.60E-03	Up
Citrate	0.66	1.035	0.680	3.87E-02	Down
beta-Ala	0.53	0.981	0.517	6.24E-05	Down
Glutathione (GSH)	0.52	8.816	4.602	2.21E-03	Down
cis-Aconitate	0.51	0.051	0.026	2.43E-02	Down
Fumarate	0.43	0.185	0.079	1.16E-02	Down
Malate	0.41	0.707	0.292	2.25E-04	Down
Glu	0.40	22.182	8.814	2.17E-04	Down
Asp	0.27	1.865	0.495	2.10E-05	Down
Ala	0.26	12.753	3.306	9.46E-05	Down
